# Adapting Human-Based Transcutaneous Spinal Cord Stimulation to Develop a Clinically Relevant Animal Model

**DOI:** 10.3390/jcm11072023

**Published:** 2022-04-05

**Authors:** Dillon C. Malloy, Maria Knikou, Marie-Pascale Côté

**Affiliations:** 1 Marion Murray Spinal Cord Research Center, Department of Neurobiology and Anatomy, Drexel University, Philadelphia, PA 19129, USA; dcm332@drexel.edu; 2 Klab4Recovery Research Laboratory, Department of Physical Therapy, College of Staten Island, New York, NY 10314, USA; maria.knikou@csi.cuny.edu

**Keywords:** spinal cord injury, transcutaneous spinal cord stimulation, neuromodulation, electrical stimulation, evoked potentials, lumbar spinal cord

## Abstract

Transcutaneous spinal cord stimulation (tSCS) as a neuromodulatory strategy has received great attention as a method to promote functional recovery after spinal cord injury (SCI). However, due to the noninvasive nature of tSCS, investigations have primarily focused on human applications. This leaves a critical need for the development of a suitable animal model to further our understanding of this therapeutic intervention in terms of functional and neuroanatomical plasticity and to optimize stimulation protocols. The objective of this study is to establish a new animal model of thoracolumbar tSCS that (1) can accurately recapitulate studies in healthy humans and (2) can receive a repeated and stable tSCS treatment after SCI with minimal restraint, while the electrode remains consistently positioned. We show that our model displays bilateral evoked potentials in multisegmental leg muscles characteristically comparable to humans. Our data also suggest that tSCS mainly activates dorsal root structures like in humans, thereby accounting for the different electrode-to-body-size ratio between the two species. Finally, a repeated tSCS treatment protocol in the awake rat after a complete spinal cord transection is feasible, tolerable, and safe, even with minimal body restraint. Additionally, repeated tSCS was capable of modulating motor output after SCI, providing an avenue to further investigate stimulation-based neuroplasticity and optimize treatment.

## 1. Introduction

Sensory and motor deficits following spinal cord injury (SCI) persist with limited functional recovery regardless of extensive efforts to optimize current therapeutic interventions. Neuromodulation strategies, in particular spinal cord stimulation, have become an increasingly popular and promising approach that can be used alone or in conjunction with well-established treatments such as locomotor training to promote functional recovery after SCI [1,2]. While animal models of epidural stimulation are abundant, transcutaneous spinal cord stimulation (tSCS), because of its noninvasive nature, has mostly been directly investigated in able-bodied and SCI individuals [3,4,5,6,7].

This has resulted in the emergence of valuable information about the feasibility and safety of this method, its access to key spinal circuitry, the benefits for patients with SCI, and its potential use in a clinical setting. Amongst the functional benefits of tSCS in SCI individuals are improvements in functional motor output [8,9,10,11,12,13,14,15,16], reduced hyperreflexia and spasticity [15,17,18,19], and improvements in volitional motor control [9,11,12,13,14]. Although critical to our understanding for therapeutic applications of tSCS, evidence-based human-only approaches are hindered by the limited number of study participants, heterogeneity of injuries, and complexity of clearly identifying neuroplastic changes in humans. The lack of knowledge on the specific neurophysiological mechanisms contributing to motor recovery with tSCS and their anatomical and molecular correlates prevents the optimization of tSCS protocols and its transition to a broader clinical population. Therefore, there is a critical need to validate a suitable preclinical animal model to further our understanding of tSCS and potentiate its use to promote functional plasticity after SCI.

In animal models, transcutaneous direct current stimulation over the thoracolumbar region has mostly been utilized [20,21,22], which is critically different from the alternated constant current and associated neuronal mechanisms of plasticity [23,24]. In the case of direct current stimulation over the thoracolumbar region, patients report discomfort and irritable sensations such as burning and tingling of the skin, and physical adverse effects include persistent skin irritations and lesions resembling burns in the area under the electrode [25,26]. In none of our applications of constant alternated current with 1 ms at 0.2 Hz did we observe blisters or burning sensation. Furthermore, the stable delivery of repeated tSCS in rodents over time has been performed at the cervical level [20,21,22,27], which has very different physiological and anatomical features, and more importantly, does not address plasticity and functional recovery in the lumbar circuitry. To our knowledge, a single study performed repeated tSCS at the thoracolumbar level in rodents, and animals were heavily restrained to ensure electrode stability [20]. The current lack of breadth in animal models for tSCS limits the progress and understanding of this intervention required to support its potential implementation into the SCI community.

In order for tSCS to optimally activate lumbar spinal neuronal networks and locomotor centers in humans, a clinically relevant animal model is in great need to allow a thorough and systematic investigation of the most effective stimulation parameters as well as identification of the mechanisms at play. The purpose of this study is to establish an effective translational approach by (1) developing a rodent model in which responses to tSCS display similar electrophysiological features as in healthy humans, and (2) determine the feasibility of delivering repeated tSCS at the thoracolumbar level in awake rats with minimal restraint and distress.

Here, we show that we were able to successfully scale down human-based tSCS to develop an animal model that activates similar neural structures and displays similar transspinal evoked potentials (TEPs) in leg muscles following tSCS as in humans. We further show the feasibility and stability of delivering tSCS over time in awake rodents, which ultimately increased the motor output of ankle extensor and flexor muscles. The validation of this model will allow us to more precisely investigate the neuroplastic changes and mechanisms of action with tSCS that otherwise cannot be learned from human studies alone.

## 2. Materials and Methods

All animal procedures were performed in accordance with the National Institutes of Health (NIH) guidelines for the care and use of laboratory animals, and experimental protocols were approved by Drexel University College of Medicine Institutional Animal Care and Use Committee. Twenty-one adult female Sprague Dawley rats (240–260 g; Charles River Laboratories, Wilmington, MA, USA) were used for all experimental procedures. Animals were housed 2–3 per cage with ad libitum food and water under 12-h light/dark cycle in temperature-controlled facilities accredited by the Association for Assessment and Accreditation of Laboratory Animal Care (AAALAC). All animals were given a one-week acclimation period upon arrival before any procedures were performed.

### 2.1. Intact Animals—Experiments

#### 2.1.1. Transcutaneous Electrode Fabrication and Stimulation Set-Up

We adapted human-based stimulation electrodes and set-up to our rat model. Re-usable, self-adhering hydrogel electrodes (Uni-Patch StarBurst Square, Balego, St. Paul, MN, USA) were cut down to 4 cm × 1 cm rectangles. Animals were briefly anesthetized with gaseous isoflurane in oxygen (1–4%), shaved over the back and abdomen, wiped clean with alcohol prep pads (70% isopropyl), and fitted with transcutaneous electrodes. One electrode (cathode) was placed over the T10-L2 thoracolumbar spine equally between paravertebral sides and no lower than the tips of the T13 floating ribs. Two of the same electrodes, connected to function as a single electrode (anode), were placed bilaterally over the abdomen. To ensure adherence and constant placement, the electrodes were covered with 3M Tegaderm transparent film (3M, St. Paul, MN, USA), and the body of the animal was lightly swaddled with self-adhesive athletic wrap (3M, St. Paul, MN, USA). This set-up was used for both the repeated tSCS treatment in SCI rats and for the terminal experiment in intact and SCI rats.

#### 2.1.2. Stimulation and Recordings

Rats (*n* = 9) were anesthetized with gaseous isoflurane in oxygen (1–4%) and fitted with transcutaneous electrodes as described above. Electromyographic (EMG) needle electrodes (Neuroline Subdermal, Ambu A/S, Ballerup, DK, USA) were placed in the left tibialis anterior (L-TA), left medal gastrocnemius (L-MG), and right tibialis anterior (R-TA) muscles, bipolar wire electrodes (Cooner Wire, Chatsworth, CA, USA) were placed in the left plantar muscles of the foot (L-Pl) on the plantar surface of the left hind paw, and ground electrodes were inserted into the skin of the arm. Rats were held at approximately 1.5% isoflurane in oxygen for the duration of the experiment.

TEPs were evoked by single monophasic 1 ms pulses through the transcutaneous cathodal electrode and recorded in L-Pl, L-MG, L-TA, and R-TA muscles. The stimulation was delivered using the DS7A constant current isolated stimulator (Digitimer Ltd., Hertfordshire, UK), which was triggered by customized scripts using the Signal software version 6.0 through a 1401 analog-to-digital data acquisition board system (CED; Cambridge Electronics Design Ltd., Cambridge, UK). EMG recordings were amplified (×1000; model 1700, A–M Systems, Sequin, WA, USA) and bandpass filtered (1 Hz–10 kHz). Signals were digitized (10 kHz) and fed to Signal software. TEPs were first recorded in response to a range of increasing stimulus intensities to construct a recruitment curve and determine motor threshold, response latency, and maximum response amplitudes for each muscle. In addition, stimulation trains (30 pulses) were delivered at 100 Hz at an intensity of 1.4 times TEP threshold (1.4 T). After completing terminal experiments, rats were sacrificed with an overdose of Euthasol (150 mg/kg, i.p.).

#### 2.1.3. Data Analysis

The recruitment curve was plotted by expressing the peak-to-peak amplitude as a function of the stimulus intensity. Peak-to-peak amplitude was measured from the maximum negative peak to the maximum positive peak within the duration of the response regardless of the number of peaks. To determine the input–output relationship, a sigmoid function (Systat SigmaPlot 14.0, Inpixon, Palo Alto, CA, USA) was fitted to individual TEP recruitment curves (i.e., each muscle of each animal) to predict maximal amplitude, slope, and stimulation intensity required to reach 50% of maximal amplitude. These parameters were used to normalize individual sigmoid functions to the predicted threshold and maximal amplitude of the curve. Group averages were calculated from the individual normalized values and used to establish a group sigmoid function.

Response latency (onset of the responses) and duration of the responses were measured at maximal amplitude as determined by the recruitment curves for each individual muscle. Latency was determined by measuring the time between stimulus and response onset, while duration was determined by the time EMG activity varied from baseline.

TEP amplitude during high-frequency stimulation trains was measured by calculating the peak-to-peak amplitude of TEPs for each of the 30 pulses and was represented as a percentage of the amplitude of the first response.

#### 2.1.4. Statistical Analysis

Significant differences between muscles were determined using a one-way analysis of variance (ANOVA) followed by the Holm–Sidak post hoc multiple comparison test unless stated otherwise. Kruskal–Wallis one-way ANOVA followed by Dunn’s post hoc multiple comparison test was used if normality or equal variance tests failed. For TEP amplitude in response to a high-frequency stimulation train, a repeated measures one-way ANOVA was utilized. Statistical analyses were performed using GraphPad Prism software version 7.04 (GraphPad Software, San Diego, CA, USA). For all tests, significance was determined when *p* < 0.05, and values are reported as the group average ± standard error of the mean.

### 2.2. Spinal Cord-Injured Animals—Procedures and Experiments

#### 2.2.1. Surgical Procedures and Postoperative Care

Rats (*n* = 12) underwent a complete spinal cord transection at the thoracic level (T10) under aseptic conditions. Gaseous isoflurane in oxygen (1–4%) was used as an anesthetic prior to and throughout the duration of the surgery. A T10–T11 laminectomy was performed, the dura was carefully split open, and the spinal cord was cut with small scissors. The completeness of the spinal transection was confirmed by examining the ventral floor of the spinal canal. Gel foam was placed into the cavity between rostral and caudal portions of the spinal cord to achieve hemostasis. Back muscles and skin incision were sutured accordingly using appropriately sized materials. Rats were singly housed for 3 days following surgical procedures before being paired for the remainder of the study. Animals received one dose of slow-release buprenorphine (0.05 mg/kg, s.c.) as an analgesic prior to surgery end and received saline (5 mL, s.c.) and Baytril (100 mg/kg, s.c.) postoperatively for 7 days to prevent dehydration and infection. Bladders were manually expressed at least twice a day for the duration of the study.

#### 2.2.2. Repeated Transcutaneous Stimulation in SCI Animals

SCI rats were randomized into one of two treatment groups: repeated transcutaneous stimulation (SCI + tSCS group, *n* = 6) or no stimulation (SCI, *n* = 6). Starting 5 days post-injury, animals from the SCI + tSCS group were fitted with transcutaneous electrodes as described above (Section 2.1.1) and secured in a modified, custom-built apparatus to allow them to lie prone with hindlimbs hanging below at rest. The motor threshold (T) was evaluated visually and with light manual touch and was determined as the lowest intensity eliciting a twitch of the ankle. Stimulations were evoked using a DS3 constant current isolated stimulator (Digitimer Ltd., Hertfordshire, UK) and a customized script written in Signal (CED). The stimulation protocol consisted of single, monophasic pulses of 1 ms in duration delivered at 0.2 Hz. Stimulation intensity alternated in bouts of 3 min between suprathreshold (1.2 T) and subthreshold (0.8 T) for a total duration of 18 min per session. Untreated SCI animals were similarly secured to the apparatus for an equal amount of time but were not stimulated. Sessions were repeated 3 times a week for 4 weeks before the terminal experiment.

#### 2.2.3. Terminal Experiments and Analysis

TEPs were elicited and recorded similar to intact animals in L-MG, L-TA, and R-TA muscles. A one-way ANOVA followed by Holm–Sidak post hoc test was run to compare latency and duration across muscles and between SCI and SCI + tSCS groups. A two-way repeated measure ANOVA followed by Holm–Sidak post hoc test was utilized to assess the effect of tSCS on TEP amplitude at increasing stimulation intensities expressed as xMT. Significance was determined when *p* < 0.05, and values are reported as the group average ± standard error of the mean.

### 2.3. Human Experiments

All procedures were performed in accordance with NIH guidelines for human research, and experimental protocol was approved by the CUNY-wide Institutional Review Board committee. Eleven healthy persons (5 males, 6 females; 24 to 34 age range) participated in the study.

#### 2.3.1. Stimulation and Recordings

We adopted similar experimental procedures as we have previously employed in humans [6]. Briefly, a self-adhering hydrogel electrode (UniPatch EP84169, 10.2 cm × 5.1 cm, Wabash, MN, USA) was placed over the T10-L2 vertebrae, and two electrodes (anode) connected to function as one were placed either over the iliac crests or abdominals based on self-reported comfort. Stimulation and recordings were performed with subjects supine, legs at midline, and hips/knees at 30° of flexion.

#### 2.3.2. Data Analysis and Statistics

In healthy humans, the recruitment curves of EMG potentials evoked by tSCS were constructed from below threshold intensities to maximal intensities that allowed for establishing motor thresholds, latencies, and maximum responses as well as the slope of the curve confined to occur at TEPs equivalent to 50% of the maximal responses. Stimulation was a single 1 ms pulse at 0.2 Hz delivered by a constant current stimulator (DS7A, Digitimer Ltd., Welwyn Garden City, UK), triggered by Spike 2 scripts through a 36-channel Power 1401 plus analog-to-digital data acquisition interface running Spike 2 (CED Ltd., Cambridge, UK). Single differential bipolar electrodes (Motion Lab Systems Inc., Baton Rouge, LA, USA) were used to record responses from ankle and thigh muscles. EMG recordings were amplified (×1000) and bandpass filtered (1 Hz–10 kHz).

A sigmoid function (Systat SigmaPlot 11, Inpixon, Plato Alto, CA, USA) was fitted to the TEPs measured as the area under the full wave rectified waveform and plotted as a function of the stimulation intensity. This was performed separately for responses recorded for each muscle and subject. Through the sigmoid function we established the m function of the slope and predicted stimuli corresponding to 50% of the maximal amplitude. These values were used to establish the slope and stimulation intensity corresponding to threshold [16]. For each muscle, the stimulation intensities and TEPs were normalized to the predicted threshold intensity and maximal amplitude, respectively. Averages of normalized TEPs for all 11 subjects were calculated in steps of 0.05 from 0.6 up to 2.5 times the stimulation threshold. Latency of TEPs was established with the cumulative sum (CUSUM) statistical method [28,29]. Each TEP was rectified and averaged, and CUSUM was applied to detect change in the series of datum points while taking into consideration 60 ms of pre-stimulation background EMG activity ± 2 standard deviation from the mean reference level [28]. The first point that the EMG signal was above the standard deviation of the EMG signal was taken as latency. Latency values are reported as the group average ± standard error of the mean.

## 3. Results

### 3.1. Adapting Electrode Configurations

**Figure 1:**
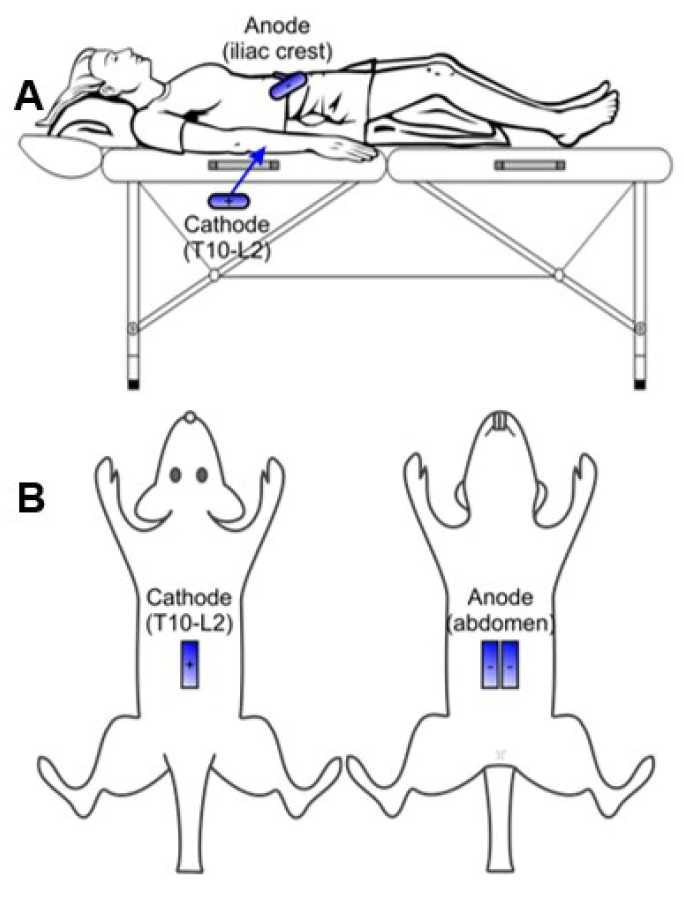
Transcutaneous electrode configuration. The cathode is placed over the T10-L2 vertebrae and the anodes over the iliac crests or abdomen in human (**A**) and rats (**B**).

We adapted human-based stimulation electrode configurations to our rat model. Reusable, self-adhering hydrogel electrodes were similarly positioned (Figure 1) and covered with Tegaderm transparent film to ensure adherence and constant placement. Electrodes were cut down from 10.2 cm × 5.1 cm for humans to 4 cm × 1 cm rectangles to fit the rat spinal cord size. The cathode was placed over the thoracolumbar spine equally between paravertebral sides over the T10-L2 vertebrae. The anode, composed of two similar electrodes, was connected to function as a single electrode and placed bilaterally over the abdomen. The animal was then lightly swaddled with self-adhesive athletic wrap.

### 3.2. TEPs Are Characteristically Similar across Muscles and between Models

**Figure 2:**
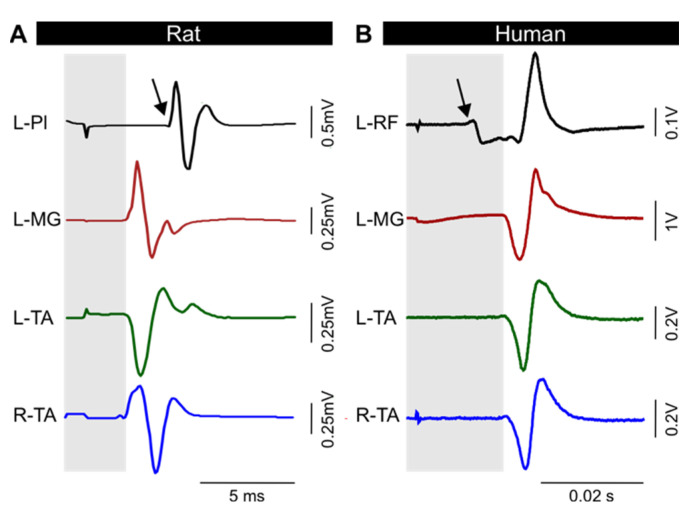
Transspinal evoked potential (TEP). Typical TEPs recorded from leg muscles in rat (**A**) and human (**B**). The response latency for ankle muscles (grey area) is different from muscle at other joints (arrow). L, left; R, right; Pl, plantar muscle; MG, medial gastrocnemius; TA, tibialis anterior; RF, rectus femoris.

To confirm the validity of our animal model, we tested the ability to produce stable and reproducible transspinal evoked potentials (TEPs) in leg muscles of intact animals following a stimulation delivered through the transcutaneous electrode [14,30,31,32,33]. As we have extensively described in humans [6], a single monophasic 1 ms duration pulse evoked responses bilaterally in rat hindlimb muscles, including the ankle flexor TA, the ankle extensor MG, and the Pl muscle (Figure 2A). Healthy humans display responses with similar characteristics (Figure 2B). The evoked responses were similar bilaterally, as depicted from the TEP recorded in the left and right TA, with similar shape and amplitude, suggesting accurate placement of the electrode at midline to ensure equal activation of the left and right motor pools. The latency of the responses recorded from a variety of hindlimb muscles ranged from 2.2 to 5.2 ms in rats and 6.8 to 22.5 ms in humans (Figure 2, see also Section 3.4). This range of latencies is consistent with spinally-induced responses. More importantly, the plantar muscle of the foot response displayed a longer latency (Figure 2A, arrow) compared to MG and TA ankle muscles in rat (grey area). This is consistent with the more caudal location of the Pl motor pool. Similarly, rectus femoris (RF) displayed a shorter latency (Figure 2B, arrow) than MG and TA in humans (grey area). Differences in latencies between different muscles and between rats and humans can be accounted for by the difference in size, conduction velocity, and location of the motor pool.

**Figure 3:**
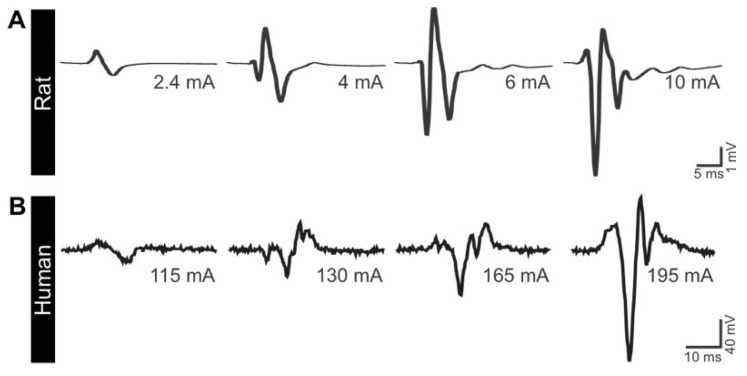
tSCS can activate complex spinal circuitry at higher intensity. As the stimulation intensity is augmented, tSCS-evoked TEPs recorded in TA display an increased number of peaks in rats (**A**) and humans (**B**).

Additionally, TEPs oftentimes displayed an increasing number of peaks with increasing stimulation intensity. An example is depicted in Figure 3. The TEPs elicited in the TA muscle were initially biphasic, with two peaks at low stimulation intensity. As the stimulation intensity increased, the number of peaks increased from 3 to 5. This suggests the ability of tSCS to activate a larger proportion of the spinal circuitry, likely including the recruitment of spinal interneurons in both rat and human models. It is worth noting that not all subjects (animals or humans) displayed this feature. While a more systematic investigation is necessary, this suggest that the excitability of motoneurons (subliminal fringe) and interneurons differs between subjects following tSCS.

### 3.3. Excitability of Motor Pools in Response to tSCS

**Figure 4:**
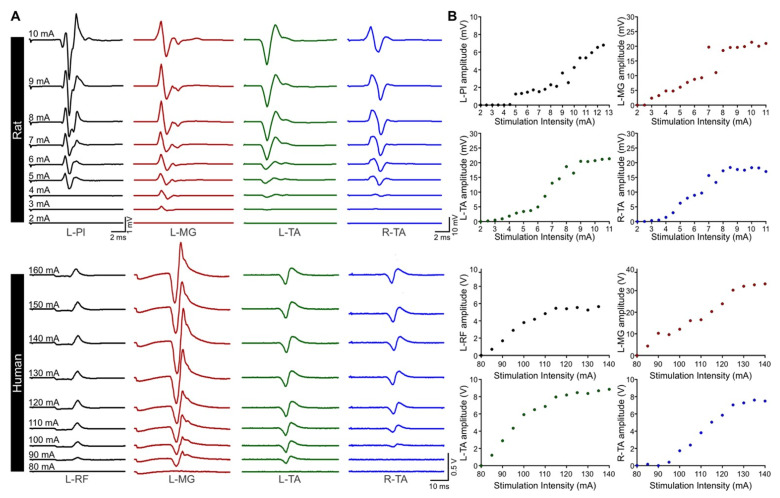
Representative examples of TEP recruitment curves. (**A**) TEPs were recorded from leg muscles at increasing stimulation intensity in a rat (**top**) and a human (**bottom**). (**B**) The amplitude of TEPs in all muscles increases with augmenting stimulaton intensity. L, left; R, right; Pl, plantar muscle; MG, medial gastrocnemius; TA, tibialis anterior; RF, rectus femoris.

We then evaluated the excitability of multisegmental motor pools innervating the legs in response to tSCS. As expected, the amplitude of TEPs increased with augmenting stimulation intensity (Figure 4A) to eventually reach a plateau at higher stimulation intensities. The recruitment curve followed a sigmoid function for all muscles recorded (Figure 4B), similar to the well-established sigmoid shape of the recruitment curves for the soleus H-reflex and M-wave, cortically-induced MEPs, and TEPs, which we have previously reported in healthy and SCI human subjects [16,34,35]. This suggests the ability of tSCS to activate motoneurons in rat hindlimbs according to the typical recruitment order of motoneurons in humans.

**Figure 5:**
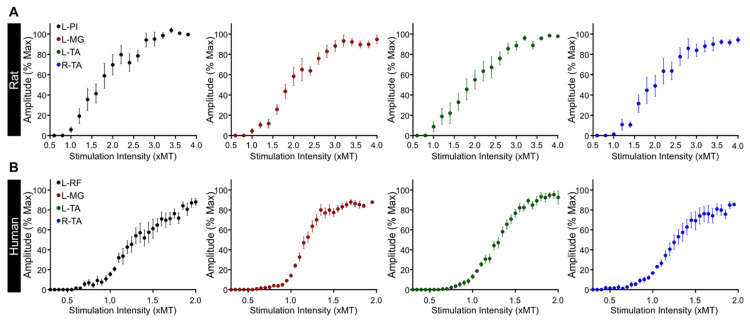
Recruitment curves of transspinal evoked potentials (TEPs). Group average of the input–output relationship in rat (**A**) and human (**B**) leg muscles. L, left; R, right; Pl, plantar muscle; MG, medial gastrocnemius; TA, tibialis anterior; RF, rectus femoris.

Overall, the recruitment of motoneurons in response to tSCS in rats (Figure 5A) is similar to that observed in humans (Figure 5B) with function of the slope m, slope, and predicted stimulation intensity corresponding to threshold, 50% of maximal, and at maximal amplitudes for each TEP (Table 1). Note the excellent sigmoid function between amplitudes and intensities, as depicted by the R^2^, and the similar parameters of TEPs recorded across different muscles.

### 3.4. Latency and Duration of TEPs

**Figure 6:**
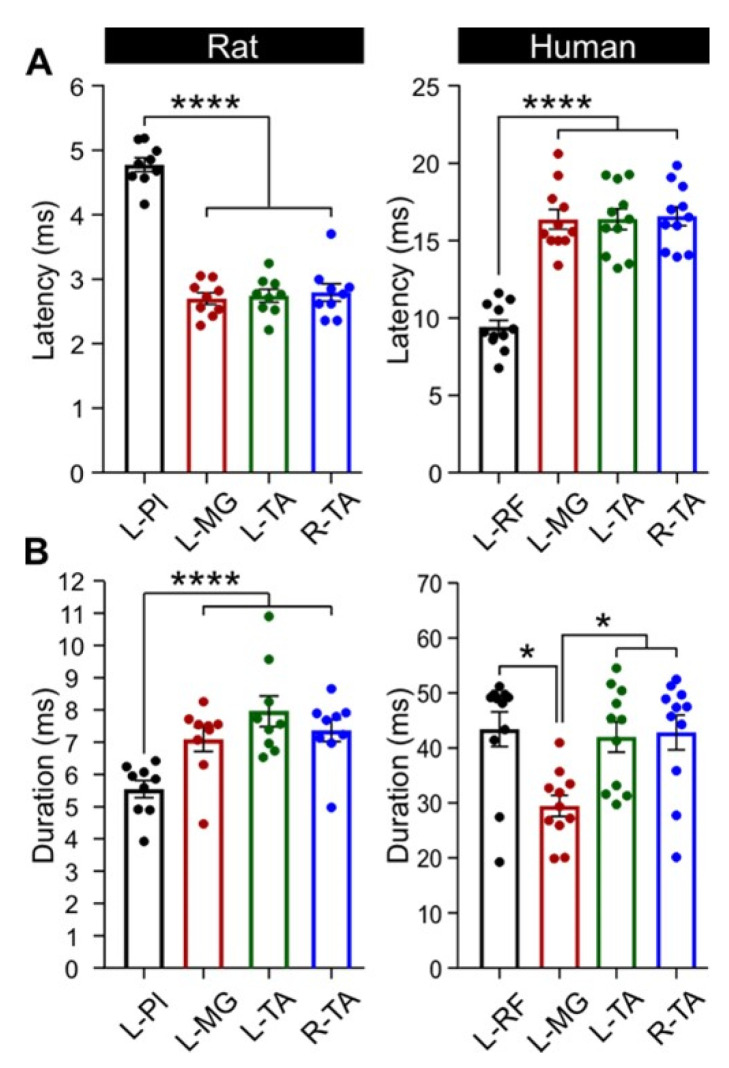
TEP latency and duration at maximal output. (**A**) L-Pl response latency was significantly longer in rats as compared to L-MG and L- and R-TA. In humans, L-RF latency was shorter as compared to ankle mucles. (**B**) In rats, the response duration was significanty shorter in L-Pl as compared to ankle muscles. In humans, MG response duration was significantly shorter than all other muscles. L, left; R, right; Pl, plantar muscle; MG, medial gastrocnemius; TA, tibialis anterior; RF, rectus femoris. * *p* < 0.05; **** *p* < 0.0001.

TEP latency (Figure 6A) was not significantly different between ipsilateral ankle flexor (L-TA, 2.74 ± 0.10 ms) and extensor muscles (L-MG, 2.70 ± 0.09 ms) or between bilateral ankle muscles (L-TA/MG and R-TA, 2.79 ± 0.14 ms; *p* > 0.05, one-way ANOVA). However, TEP onset latency for the L-Pl muscle was significantly delayed (4.78 ± 0.11 ms) compared to ankle muscles (F_3,32_ = 86.7, *p* < 0.0001, one-way ANOVA). This is in agreement with the more distal location of the Pl muscle and caudal location of the motor pool as compared to more proximal muscles such as the TA and MG. Similarly, TEP onset latency in humans was clearly shorter for proximal thigh muscles (RF, 9.41 ± 1.49 ms) as compared to distal ankle muscles (TA, 16.49 ± 2.12 ms and MG, 16.37 ± 2.11 ms; Figure 6A). One-way ANOVA showed that the latency was significantly different across muscles (F_4,50_ = 32.607, *p* < 0.01). Holm–Sidak multiple comparisons showed that the latency of L-RF was significantly different from ankle muscles (*p* < 0.001), while the latency of ankle flexors and extensors was similar (*p* > 0.05).

The response duration (Figure 6B) at maximal TEP amplitude was not significantly different between ipsilateral or bilateral ankle muscles (L-TA, 7.96 ± 0.48 ms; L-MG, 7.09 ± 0.37 ms; R-TA, 7.36 ± 0.34 ms) (*p* > 0.05, Kruskal–Wallis one-way ANOVA). The response duration of L-Pl TEP (5.54 ± 0.27 ms) was, however, significantly less than ankle muscles (H (3) = 16.08, *p* < 0.05, Kruskal–Wallis one-way ANOVA). This was predictable, as this is a small muscle which has a limited number of muscle fibers within the plantar surface of the hind paw. It is noteworthy that similar results were obtained when latency and duration was measured at oui 30% of the TEP maximal response (not shown). In humans, TEP duration measured at maximal stimulation intensities (Figure 6B) was different between L-RF and L-MG as well as between R-TA and L-MG and between L-TA and L-MG (H (3) = 19.71, *p* < 0.05, Kruskal–Wallis one-way ANOVA on ranks), suggesting that more interneurons are recruited for responses recorded from ankle and hip flexors following cathodal thoracolumbar tSCS in healthy humans.

### 3.5. Rat tSCS Activates Primary Afferents

**Figure 7:**
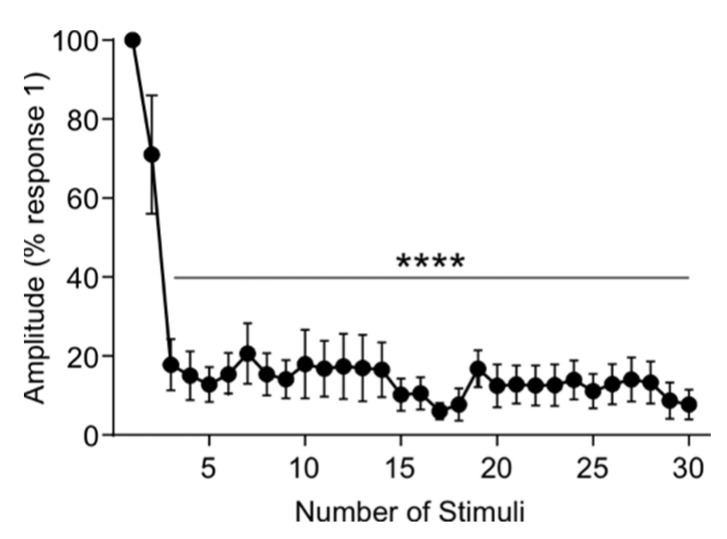
High-frequency pulse train with tSCS. TEP amplitude was recorded in response to a 30-pulse train of high frequency (100 Hz) tSCS at suprathreshold (1.4 T, motor threshold) intensity. TEP amplitude was significantly decreased compared to pulse number 1 from pulses 3 through 30. **** *p* < 0.0001.

To confirm that tSCS in rats is mostly mediated by primary afferents as reported in humans [4,36,37,38,39,40], we measured the latency and amplitude of TEPs induced by high-frequency stimulation trains (30 p, 100 Hz, 1.4 T). The onset latency of TEPs was similar across all 30 pulses (Kruskal–Wallis one-way ANOVA, H (29) = 16.572, *p* = 0.968) with an average of (5.19 ± 0.23 ms), and no different from TEPs evoked by single pulse (Figure 6A, 4.78 ± 0.11). As expected from the repetitive stimulation of primary afferents in rats [41,42] and humans [43], TEP amplitude decreased with the second response at 71.05 ± 15.04% of the first response, and all subsequent responses continually showed significant depression (Figure 7; F_3,18_ = 18.42, *p* < 0.0001, one-way ANOVA) as compared to the first response with an average amplitude of 13.58 ± 5.49%. This suggests that tSCS in our rat model is also, at least partly, mediated by the activation of primary afferents.

### 3.6. Repeated tSCS in the Awake Rat Is Feasible, Tolerable, and Safe

**Figure 8:**
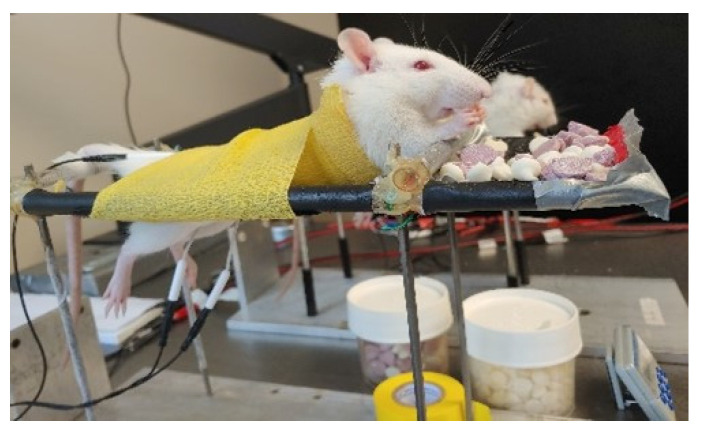
Transcutaneous spinal cord stimulation in the awake rodent.

After validating the similarity between tSCS responses in neurologically intact rats and humans, we tested the possibility to deliver tSCS as a treatment in awake SCI animals. We were able to test the feasibility, tolerability, and safety of tSCS in awake animals by providing chronic, repeated treatment 3 days per week for 4 weeks. By securing rats in a custom-built apparatus in a physiologically normal resting state with hindlimbs hanging below at rest (Figure 8), we were able to stimulate animals without movements or changes in body position that would jeopardize the placement of the transcutaneous electrodes. This was confirmed by measuring the stimulation intensity required to reach motor threshold over time, where intensity was consistent throughout and not significantly different from the first session (not shown). This suggests that the placement of the transcutaneous electrode was consistent and stable over time with repeated placement. The stimulation protocol was well-tolerated for the 18 min duration of each treatment session. In addition, animals did not show any visual signs of pain or discomfort during stimulation bouts whether the stimulation intensity was above or below motor threshold, including but not limited to vocalization, wincing, orbital tightening, or ear folding [44]. Therefore, repeated tSCS in the awake rat proved to be feasible to perform and reproduce in a chronic setting, tolerable throughout each repeated treatment session over time, and safe with minimal risk of tissue damage or discomfort similar to humans.

### 3.7. Repeated tSCS Increases Motor Output in Ankle Muscles of the Spinalized Rat

**Figure 9:**
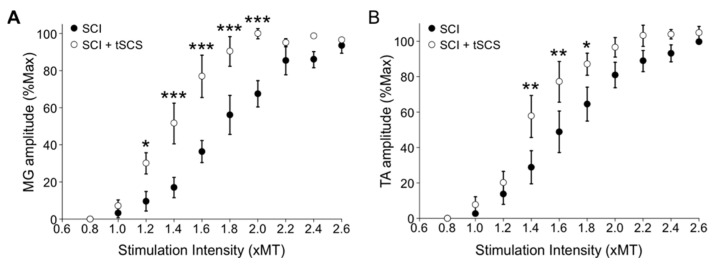
Repeated tSCS increases motor output after SCI. The recruitment curves of TEPs recorded in MG (**A**) and TA (**B**) show that the amplitude is larger in SCI + tSCS than SCI. * *p* < 0.05, ** *p* < 0.01, *** *p* < 0.001.

We have recently shown that repeated tSCS increases motor output based on TEP recruitment curves in people with motor complete or incomplete SCI [16]. To investigate the potential of repeated tSCS to produce similar results in spinalized animals, we built TEP recruitment curves for the MG and TA muscles, as constructed in intact animals (Figure 4 and Figure 5). MG and TA TEPs were similar in shape, latency, and duration whether the animals had received tSCS or not (*p* > 0.05, one-way ANOVA, not shown). Response latency and duration for SCI animals were 2.66 ± 0.23 ms and 5.82 ± 0.61 ms (MG) and 2.75 ± 0.14 ms and 5.99 ± 0.32 ms (TA), respectively, and were 2.51 ± 0.15 ms and 5.23 ± 0.35 ms (MG) and 2.72 ± 0.15 ms and 5.51 ± 0.20 ms (TA), respectively, for SCI + tSCS animals. A two-way repeated measure ANOVA revealed an interaction between groups (SCI vs. SCI + tSCS) and stimulation intensities (from 0.8 to 2.6 in 0.2 xMT increments) for both MG (F_10,81_ = 5.5190, *p* <0.001) and TA (F_10,109_ = 2.165, *p* = 0.025). A Holm–Sidak post hoc test identified that repeated tSCS significantly increased TEP amplitude in the MG at intensities ranging from 1.2 to 2 T (Figure 9A) and in the TA from 1.4 to 1.8 T (Figure 9B) as compared to untreated SCI animals. These results further support that the actions of tSCS include increased motor output after SCI. This also indicates that repeated tSCS in SCI rats can recapitulate human-based treatment paradigms and produce similar findings as in SCI humans after tSCS [16,17].

## 4. Discussion

The development and application of clinically relevant animal models for noninvasive tSCS are paramount to understand this therapeutic intervention at the neuroanatomical level. However, it is necessary that these animal models have similarities to human-based tSCS for adequate comparison and extrapolation of results between the two species.

Appropriately scaling down tSCS to rats had certain challenges. The selectivity of tSCS is primarily determined by spatial conditions, especially the design and the placement of stimulating electrodes. Spatial selectivity is also strongly limited by the distance between the electrode and target neuron, the diverse distribution of tissue conductivity, and the resulting distribution of the electrical field [5,45,46,47]. Importantly, nonhomogeneous electrical conduction properties, such as bony structures of the spine, also differ between rats and humans and could have a significant influence on the generated electrical fields. Considering this and the different electrode-to-body-size ratio in rats as compared to humans, it was critical to ensure that the same neural structures were stimulated with tSCS in rats and that TEPs displayed similar neurophysiological characteristics.

While the neuronal circuits and pathways activated by tSCS are not yet fully understood, it is believed to activate similar neuronal structures as epidural stimulation [40]. It has been suggested to mainly excite primary afferents in the dorsal roots leading to transsynaptic excitation of motoneurons and spinal interneuronal networks over multiple spinal segments close to and far from the stimulation site [4,36,37,38,39]. However, the presence of orthodromic and antidromic volleys traveling across the mixed peripheral nerve needs to be determined given the summation of soleus H-reflex and soleus TEP action potentials on surface EMGs as well as the depression of soleus H-reflex or soleus TEP based on the relative timing between the two stimuli [48]. As expected from the stimulation of primary afferents, the amplitude of TEPs evoked by a high-frequency train of tSCS was significantly depressed in intact rats. This response contrasts with direct motoneuronal stimulation, which would either cause facilitation or lack of depression [49,50]. Finally, if the conduction of the electrical current was extended through soft tissues located outside the spinal networks, delayed EMG responses would have been expected but were not observed.

The similar recruitment curves in rat hindlimb muscles and healthy human leg muscles (Figure 5) following tSCS support an orderly recruitment of motoneurons. The net system gain may thus be estimated from the slope of the sigmoid function, which, in turn, can potentially reflect activation of motoneurons residing within the subliminal fringe. Transcutaneous spinal cord stimulation generated TEPs in bilateral hindlimb muscles at different joints with biphasic, triphasic, and polyphasic waveforms at increasing stimulation intensities that are characteristics of responses evoked in human leg muscles [5,6,36,48]. The increasing number of phases at increasing intensities suggest the activation of spinal interneurons, as supported from the polysynaptic effects on soleus H-reflex when it is evoked at its maximal amplitude [48]. We also observed a preferential activation of proximal and distal motor pools based on their location along the lumbar spinal cord in both rats and humans, with distal limb muscles having longer latencies compared to those more proximal to the stimulation site. This is comparable between the two species when corrected for distance traveled and conduction velocity differences [6,51,52,53,54,55]. Lastly, we should note that more research is needed to determine whether the maximal TEP reflects depolarization of the whole motoneuron pool, as is the case for the maximal M-wave [56].

Since the development of this model is ultimately meant to be used as a treatment after neurological injuries, we tested a protocol in awake SCI animals using the same stimulation configuration as initially used in intact animals during terminal experiments. Throughout the stimulation session, the animals were secured to a custom-built support harness and displayed no discomfort whether the stimulation intensity applied was above or below TEP threshold. We have more than 10 years of experience using this type of restraint in SCI rats for bicycling up to 1 h per day with no issue [57,58,59]. This is significant progress in delivering a clinically relevant treatment that does not necessitate anesthesia or immobilization of the animal [20,60].

We further investigated the effect of repeated tSCS over 4 weeks using alternated subthreshold and suprathreshold 0.2 Hz stimulation over the lumbar enlargement of rats with a complete SCI. This protocol was prioritized to match that used in humans by Knikou in both able-bodied and SCI individuals [16,17] and confirm that the effects of repeated tSCS can be reproduced in a rodent model of complete transection. Repeated tSCS increased net motor output, as assessed by TEP recruitment curves, supporting a similar effect in rats and SCI individuals [16]. Now that we have validated the feasibility of repeated tSCS stimulation in a rodent model and its relevance to humans in terms of spinal excitability, it will allow us to move forward and utilize this treatment intervention of tSCS in animals with a contusion injury. Our next objective is to use tSCS in anatomically incomplete spinal rats to further bolster the clinical relevance of this model across both intervention and injury.

Together, we have provided compelling evidence that our rat model shares common neurophysiological characteristics with similar physiological properties following tSCS to those observed in human subjects. We have also demonstrated that repeated stimulation can be performed in the awake SCI animal and that this treatment increased motor output in both flexor and extensor muscles of the hindlimbs. This strongly supports that our animal model can be effectively utilized to further our understanding of neuroplasticity induced by tSCS after SCI and how it affects functional recovery. This will be instrumental to optimize this therapeutic intervention and accelerate its transition to the clinic. 

## Figures and Tables

**Table 1:** Parameters of sigmoid function for the TEP recruitment curves in intact animals and healthy humans.

		R^2^	Max	m	S50	Slope	S-Threshold	S-Max
Rats	L-Pl	0.958 ± 0.013	2.29 ± 1.03	1.08 ± 0.21	6.03 ± 0.92	2.36 ± 0.46	4.21 ± 0.55	8.94 ± 1.27
	L-MG	0.977 ± 0.003	14.62 ± 2.12	1.36 ± 0.32	5.17 ± 0.39	2.00 ± 0.31	3.17 ± 0.24	7.18 ± 0.66
	L-TA	0.984 ± 0.004	12.76 ± 2.85	1.42 ± 0.21	4.39 ± 0.48	1.59 ± 0.16	2.80 ± 0.41	5.98 ± 0.59
	R-TA	0.986 ± 0.003	14.94 ± 2.63	2.13 ± 0.60	4.35 ± 0.60	1.45 ± 0.31	2.91 ± 0.38	5.80 ± 0.87
								
Humans	L-RF	0.923 ± 0.018	7.90 ± 1.94	0.08 ± 0.03	170.84 ± 14.07	52.37 ± 14.82	118.47 ± 10.88	223.21 ± 36.64
	L-MG	0.947 ± 0.013	20.17 ± 2.46	0.08 ± 0.02	175.95 ± 14.07	31.56 ± 4.76	144.39 ± 11.56	207.51 ± 17.53
	L-TA	0.953 ± 0.005	7.16 ± 0.83	0.05 ± 0.01	170.63 ± 13.08	53.66 ± 8.82	116.97 ± 9.27	224.28 ± 20.29
	R-TA	0.950 ± 0.010	9.68 ± 1.52	0.05 ± 0.01	177.73 ± 16.29	58.01 ± 9.98	119.71 ± 11.12	235.74 ± 24.62

Average ± SEM predicted values from the sigmoid fit with stimulation intensities plotted against TEP amplitude. Max is the maximal amplitude of the TEP; m is the slope parameter of the sigmoid function; S50 is the stimulus intensity required to elicit a TEP equivalent to 50% of the maximal amplitude (mA); slope is the slope of the sigmoid relationship confined to occur at S50; S-threshold and S-max are predicted stimulation intensities (mA) corresponding to threshold and maximal amplitudes, respectively. L, left; R, right; Pl, plantar muscle; MG, medial gastrocnemius; TA, tibialis anterior; RF, rectus femoris.

## Data Availability

The data presented in this study are available on request from the corresponding author.
